# Expedited COVID-19 vaccine trials: a rat-race with challenges and ethical issues

**DOI:** 10.11604/pamj.2020.36.206.23977

**Published:** 2020-07-22

**Authors:** Prathamesh Haridas Kamble, Siddharth Pramod Dubhashi

**Affiliations:** 1 Department of Physiology, All India Institute of Medical Sciences, Nagpur, India; 2 Department of General Surgery, All India Institute of Medical Sciences (AIIMS), MIHAN, Nagpur, India

**Keywords:** COVID-19, vaccine, ethics

## Abstract

The intense global efforts are directed towards development of vaccines to halt the COVID-19 virus pandemic. There are 160 candidate vaccines under clinical trials across the world using different molecular targets and techniques. This race for the vaccine has several challenges and ethical issues like compressed timelines, generation and proper management of resources and finances, risks to the participating volunteers due to curtailed research trial processes, geopolitical contentions, misinformation through social media and parallel race with drugs. We feel that the fundamental principles of ethics: autonomy, beneficence, non-maleficence and justice should not be violated in this hastened vaccine development process. We recommend constitute a Consortium on a global platform to formulate, provide and monitor a comprehensive ethical umbrella to the process of vaccine development.

## Perspective

**Figure 1:**
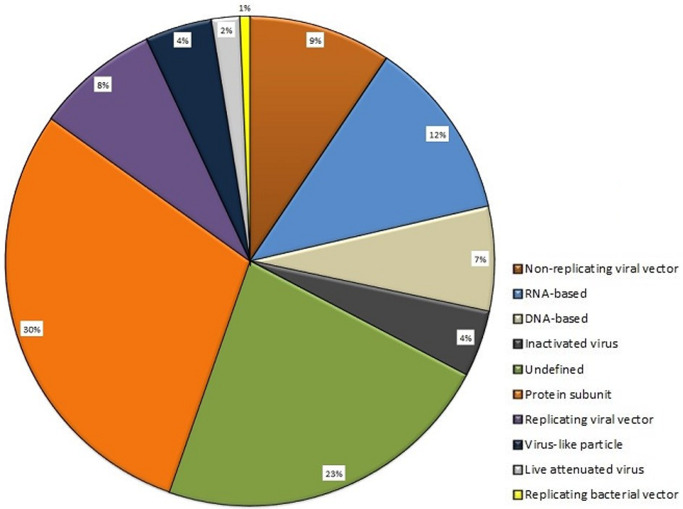
candidate vaccines

The COVID-19 pandemic due to the highly contagious SARS-CoV-2 virus has taken the world by storm. In this regard, countries all over the world are devising various policies and processes like travel restrictions, social restrictions, work from home orders, curfews, lockdowns, and contact tracing for confirmed cases. But eventually, these strategies may not be effective, when lockdowns are relaxed. The only ray of hope is development of COVID-19 vaccine on priority. The genetic sequence of the coronavirus, SARS-CoV-2, published on 11 January 2020 by China, opened up a new horizon for the possible vaccine to prevent the global outbreak of COVID-19. In response to this, intense global efforts were directed towards development of vaccines with shortened timelines. More than 160 vaccine candidates are under clinical trials across the world. A number of molecular targets have been tried and tested. Several molecular platforms have been identified for vaccine production as shown in Figure 1 [1]. The details of some of the promising vaccine developments are summarized in Table 1 [1]. COVID-19, a novel virus with details getting updated every day, new viral targets are being discovered and many are under preclinical research study. Table 2 depicts few of the potential vaccines which are in preclinical studies and potential to start Phase I trials [1]. Even though rapid, promising and innovative global efforts are being made towards development of COVID-19 vaccine, there are several challenges and issues involved:

**Table 1:** landscape of several promising vaccine candidates under phase I & II clinical trials

Vaccine candidate	Technology used	Clinical trial phase	Country of origin	Month of initiation
Ad5-nCoV	recombinant adenovirus type 5 vector	Phase II	China	March 2020
Ad5-nCoV	recombinant adenovirus type 5 vector	Phase I	China	March 2020
ChAdOx1 nCoV-19	Adenovirus	Phase II	UK	April 2020
BNT162 (a1, b1, b2, c2)	RNA	Phase II	Germany US	April 2020
INO-4800	DNA plasmid delivered by electroporation	Phase II	US South Korea	April 2020
mRNA-1273	lipid nanoparticle dispersion containing messenger RNA	Phase I	US	March 2020
bacTRL-Spike	DNA, bacterial medium (oral)	Phase I	Canada	April 2020
NVX-CoV2373	SARS-CoV-2 recombinant spike protein nanoparticle with adjuvant	Phase I	Australia	May 2020

**Table 2:** promising vaccine candidates which are under preclinical studies

Vaccine candidate	Technology used	Vaccine candidate	Technology used
DPX-COVID-19	protein subunit, lipid-based delivery	PittCoVacc	protein subunit, microneedle arrays
CureVac	RNA, mRNA	LUNAR-COV19	RNA, mRNA
CoroFlu	self-limiting influenza virus	AdCOVID	non-replicating viral vector; intranasal
Unnamed	non-replicating viral vector; oral	Unnamed	pan-coronavirus

**Non-specific vaccine:** BCG, Measles -Mumps-Rubella vaccine

**Compressed Timelines**: the standard vaccine development cycle, including research & development (phase I to IV of clinical trials), manufacturing, approvals, licensing, distribution and deployment take several years. But the COVID-19 pandemic storm has compressed the timelines of coronavirus vaccine development to a few months. There are several obstacles to meet these shortened deadlines: geopolitical issues, international politics and competitions, social distancing and guidelines mandating closedown of laboratories across globe, optimum manufacturing of billions of doses and licensing, and quick global distribution of vaccines. The major threat to the compressed timeline is curtailment and compromise in different steps of research and development. Counter measures: The governments, researchers and manufacturers are adopting the ‘fast-tracking the testing and regulatory review of vaccines’ policies. The safety and efficacy phases of trials are running parallel to save on time, instead of the traditional sequential protocols. Many manufacturing companies have started mass production of vaccines accepting the risk of failure of vaccines during trials. Others are working with regulators in multiple countries simultaneously, looking for the quickest path to market. On similar lines, the US government has launched a ‘Operation Warp Speed’ program to fast track the development and mass manufacturing phase of eight diverse vaccine candidates with a deadline of January 2021 [2].

**Resources and Finances:** in the history of humanity, a vaccine for infectious diseases had never been produced in several years. The expedited development of COVID-19 vaccine not only needs mammoth resources and funds, but also an effective monitoring system to check wastage of the same. Counter measures: Several Organizations have been formed for international collaboration: Coalition for Epidemic Preparedness Innovations (CEPI) have catered a global tie up between public, private, civil societies and non-governmental charitable trusts to expedite the research and development of eight candidate vaccines catering 2 billion US dollars [3]; The WHO through a telethon raised 8 billion US dollars to support the vaccine development process; Gates Foundation donated 250 million US dollars for research and distribution of vaccines; Global Alliance for Vaccines and Immunization (GAVI) is financing 200 million US dollars [4]. Moreover, to monitor and coordinate multiple vaccine clinical trials across the world, WHO announced the ‘Solitary Trial’ for international deployment of several simultaneous vaccine trials, for rapid interpretation and sharing results, simultaneously evaluating risk-benefit ratio, prioritizing the vaccines to be taken into next phase and avoiding duplication of research efforts [5].

**Future distribution of the eventual vaccine:** amongst all the Individual countries developing or producing COVID-19 vaccine may be persuaded to favor their own country as the funding comes from taxpayers. This may deprive the low economic countries where there is actually an urgent need. Moreover, the vaccine should be available on priority to health care workers and high-risk groups. The pharmaceutical companies manufacturing vaccines should make it affordable to all populations keeping aside their own benefits [6]. Counter measure: Several research universities, manufacturers and collaborations have consented to distribute the developed vaccine multinational in cooperation with WHO and to manufacture vaccines at a non-profitable basis for early stages to meet the needs of the society. The WHO & CEPI have drafted their guidelines for global deployment of vaccines depending upon the needs of the country and population at risk [7]. *Risks involved in hastened vaccine development research:* the rapid development and urgency of producing a vaccine for the COVID-19 pandemic may increase the risks and failure rate of delivering a safe, effective vaccine. The vaccine may have unintended side effects due to suboptimal antibody both in terms of quality and quantity, leading to antibody-dependent disease enhancement (ADE) [8]. Many transgenic strains of animals (e.g. ACE-2 transgenic mice) have been bred for COVID vaccine preclinical phase; they may pose a threat to emerge as a new disease, if proper biosafety measures are now followed in the race to win the vaccine. Several ethical concerns [9] have been raised regarding the proposed ‘challenge study’ [10] models wherein to accelerate the path to licensing the conventional the Phase III trial will be curtailed or modified and the healthy volunteers are deliberately exposed to risk of infection by injecting trial vaccine. To regulate this, WHO has developed guidance document setting in several criteria for the ethical acceptability of COVID-19 human challenge studies [11].

**Global race for vaccine:** due to ever growing sense of nationalism, both the pride and profit at stake, the race and contention for first to develop a vaccine is already heated up. The raw nerves in the relationship between the different countries were exposed. This ever growing simmering global tension over the race may pose a serious threat to global peace and harmony in times to come. This race has further percolated in the pharmaceutical industries and manufacturers where they are sinking billions into a bet with a timid chance of success. If successful, they will expect hefty financial returns making affordability to the overall population questionable. To counteract this issue, some giants in the pharmaceutical industry have promised to make vaccines available at low cost at first and they may reap up the cost if vaccines are needed for seasonal use and for stocking up by any nation in future.

**Misinformation through social media:** world is already fighting an epic battle against the coronavirus misinformation; 4G/5G towers are fueling corona spread, drinking bleaches and injecting UV rays can cure coronavirus, and the coronavirus conspiracy theory claiming the virus behind the COVID-19 was already known and vaccine is available, are just to name a few of them. Tracking them and curbing their spread is really challenging due to widespread use of social media. Such misinformation may unusually be effective in sowing doubts about COVID vaccine as being sinister rather than a savior. It may be perceived as unsafe and unnecessary leading to vaccine hesitancy (refusing vaccine) which will further increase the future risk of COVID-19 outbreaks.

**Vaccine vs drugs:** while the vaccines take time for thorough testing, the potential drug development is making incremental advances and has jumped into the COVID vaccine race. Some of these drugs are targeted towards stopping the viral replication, while some are helping to calm the exaggerated immune response and organ damage. The advantage of drugs over vaccines is that results are seen quickly as they are tested in already sick patients, unlike the vaccines, wherein researchers have to monitor the infection potential. The vaccine needs to be extremely safe as it is given in healthy subjects compared to drugs, which may have acceptable risk in already sick patients. The repurposing of drugs can be another effective way to make COVID-19 less fatal and/or morbid. The early results of drugs like Remdesivir [12] are encouraging and highlight the drugs as front-runners in the race.

**Recommendations:** 1) The fundamental principles of ethics: autonomy, beneficence, non-maleficence and justice need to be applied to the process of vaccine development. 2) A COVID-19 Vaccine Consortium on a global platform, which would provide a comprehensive ethical umbrella to the process of vaccine development, is the need of the hour. 3) The said Consortium should include clinicians, immunologists, epidemiologists and ethicists. 4) A concerted effort towards formulating a definite scientific rationale behind human challenge studies is essential, especially in the context of understanding of pathophysiology of Covid-19 still being in a fluid state. 5) The Consortium also needs to formulate strategies for resource allocation and prioritization for vulnerable groups.

## References

[1] Thanh Le T, Andreadakis Z, Kumar A, Gómez Román R, Tollefsen S, Saville M (2020). The COVID-19 vaccine development landscape. Nat Rev Drug Discov.

[2] Cohen J (2020). U.S 'Warp Speed' vaccine effort comes out of the shadows. Science.

[3] UN News 'Landmark collaboration' to make COVID-19 testing and treatment available to all.

[4] GAVI: The Vaccine Alliance COVID-19: Gavi steps up response to pandemic.

[5] World Health Organisation (2020). Accelerating a safe and effective COVID-19 vaccine. Accessed 2 June.

[6] Gates B (2020). Responding to Covid-19-A Once-in-a-Century Pandemic?. N Engl J Med.

[7] Yamey G, Schäferhoff M, Hatchett R, Pate M, Zhao F, McDade KK Ensuring global access to COVID-19 vaccines. The Lancet. 2020;.

[8] Iwasaki A, Yang Y (2020). The potential danger of suboptimal antibody responses in COVID-19. Nat Rev Immunol.

[9] Callaway E (2020). Should scientists infect healthy people with the coronavirus to test vaccines?. Nature.

[10] Eyal N, Lipsitch M, Smith PG (2020). Human Challenge Studies to Accelerate Coronavirus Vaccine Licensure. J Infect Dis.

[11] World Health Organization Key criteria for the ethical acceptability of COVID-19 human challenge studies. Global health ethics.

[12] National Institutes of Health (NIH) NIH clinical trial shows Remdesivir accelerates recovery from advanced COVID-19.

